# Sulfate-mediated Drought Tolerance in Maize Involves Regulation at Physiological and Biochemical Levels

**DOI:** 10.1038/s41598-020-58169-2

**Published:** 2020-01-24

**Authors:** Muhammad Munir Usmani, Fahim Nawaz, Sadia Majeed, Muhammad Asif Shehzad, Khawaja Shafique Ahmad, Gulzar Akhtar, Muhammad Aqib, Rana Nauman Shabbir

**Affiliations:** 1 Department of Agronomy, MNS University of Agriculture, Multan, Pakistan; 2 Department of Agronomy, University College of Agriculture and Environmental Sciences, Bahawalpur, Pakistan; 3 Department of Botany, University of Poonch, Rawalakot, 12350 Pakistan; 4 Department of Horticulture, MNS University of Agriculture, Multan, Pakistan; 5 Department of Agronomy, Faculty of Agriculture, Bahauddin Zakariya University, Multan, Pakistan

**Keywords:** Plant physiology, Drought

## Abstract

Restriction in nutrient acquisition is one of the primary causes for reduced growth and yield in water deficient soils. Sulfur (S) is an important secondary macronutrient that interacts with several stress metabolites to improve performance of food crops under various environmental stresses including drought. Increased S supply influences uptake and distribution of essential nutrients to confer nutritional homeostasis in plants exposed to limited water conditions. The regulation of S metabolism in plants, resulting in synthesis of numerous S-containing compounds, is crucial to the acclimation response to drought stress. Two different experiments were laid out in semi-controlled conditions to investigate the effects of different S sources on physiological and biochemical mechanisms of maize (*Zea mays* L. cv. P1574). Initially, the rate of S application in maize was optimized in terms of improved biomass and nutrient uptake. The maize seedlings were grown in sandy loam soil fertigated with various doses (0, 15, 30 and 45 kg ha^−1^) of different S fertilizers viz. K_2_SO_4_, FeSO_4_, CuSO_4_ and Na_2_SO_4_. The optimized S dose of each fertilizer was later tested in second experiment to determine its role in improving drought tolerance of maize plants. A marked effect of S fertilization was observed on biomass accumulation and nutrients uptake in maize. In addition, the optimized doses significantly increased the gas exchange characteristics and activity of antioxidant enzymes to improve yield of maize. Among various S sources, application of K_2_SO_4_ resulted in maximum photosynthetic rate (43%), stomatal conductance (98%), transpiration rate (61%) and sub-stomatal conductance (127%) compared to no S supply. Moreover, it also increased catalase, guaiacol peroxidase and superoxide dismutase activities by 55, 87 and 65%, respectively that ultimately improved maize yield by 33% with respect to control under water deficit conditions. These results highlight the importance of S fertilizers that would likely be helpful for farmers to get better yield in water deficient soils.

## Introduction

Maize (*Zea mays* L.) is one of the major cereal crops that provide food for humans and feed for livestock. The demand for maize seed has increased significantly during past few decades due to its consumption in poultry feed and wet milling industry. The importance of maize as a food crop is well recognized and is used as a staple food in many parts of the world^1^. Maize seed is an abundant source of energy as 100 g seed provides 365 kilocalories of energy^2^. However, it is an extensive nutrient crop and excessive use of fertilizers to obtain high yield has resulted in the depletion of nutrients particularly sulfur (S) in soils^3^. Moreover, water shortage due to climate change may induce further losses in maize production in future.

Adaptation of maize to limited water conditions has received great interest from farmers, researchers and policy makers considering its importance in nutritional food security. Since maize requires large quantities of water to complete its life cycle, water deficiency at critical growth stages significantly reduces maize yield^4^. Exposure to drought stress induces marked changes in water status, chlorophyll content and photosynthetic apparatus of plants^5^. It influences water use efficiency and biomass accumulation in plants^6^. Drought induced reduction in nutrients absorption, redistribution and transport adversely affects the plant production^7,8^. Due to utilization of large amount of nutrients, maize is considered sensitive to nutrient deficiency^3^, which may be further aggravated by limited water conditions. Increased deficiency of nutrients in agricultural soils is considered one of the major factors for reduced maize yield^9^.

Sulfur (S) is recognized as the fourth major nutrient after nitrogen (N), phosphorus (P) and potassium (K). It not only improves crop yield but also influences the quality due to its key role in protein synthesis. It is main constituent of proteins, thioredoxin (TRx), methionine (Met), cysteine (Cys), vitamins (Vit), sulfo-lipids (SL) and iron-sulfur (Fe-S) clusters system that play important role in regulation of physiological metabolism of plants^10^. Plants uptake S in metabolically inactive form known as sulfate (SO_4_^2−^) from soil surface. It is reduced into sulfide (S^−2^) and assimilated into cysteine (Cys) by the activity of ATP-sulfuryase^11^. A variety of S compounds such as glutathione (Glu), Met, phytochelatins (PCs) are synthesized from Cys-residues which play an important role in alleviating the drastic effects of environmental stresses like drought^12^. Interestingly, S is the only macronutrient that accumulates in the xylem sap of water stressed maize plants^13^. Recent research suggests a coordinated action of several drought-responsive stress metabolites with S assimilation in plants exposed to drought stress^14^. For example, abscisic acid (ABA) induced closure of stomata is directly linked with S metabolism in maize^13^. The increased S demand in drought-stressed plants reflects the regulatory importance of S in ABA signalling and detoxification of reactive oxygen species (ROS)^15^. The highly reductive glutathione or GSH scavenges ROS such as OH^•^, O^•−^_2_ and H_2_O_2_ through activation of enzymatic antioxidants viz. catalase (CAT), guaiacol peroxidase (GPX) and superoxide dismutase (SOD). Both CAT and GPX eliminate excess H_2_O_2_ by generating H_2_O and O_2_, whereas SOD prevents OH^•^ formation by removing O^•−^_2_^16,17^. Additionally, S metabolism is linked to polyamine and ethylene through salvage pathway involved in maize response to drought stress^3,11^.

Studies involving the use of S fertilizers to improve crop produce and productivity are well documented^18–22^. However, the questions pertaining to comparative effects of S fertilizers on uptake and metabolism of other nutrients have largely remained unanswered. It is momentous to unravel the effects of S on mineral elements particularly nitrogen (N), potassium (K) and phosphorus (P). Evidence suggests that decrease in SO_4_^2−^ availability during drought may restrict nitrate (NO_3_^−^) uptake due to limited CO_2_ fixation and decreased flux of SO_4_^2−^ into cysteine in maize^11^. Therefore, a balanced N:S ratio is essential to obtain high yield and quality in cereal crops^23^. Remarkably, rhizospheric S availability regulates K content in shoot indicating that K^+^ acts as counter-ion to compensate for SO_4_^2−^ deficiency in soil^24^. Likewise, remobilization of vacuolar SO_4_^2−^ during S deprivation was compensated osmotically by accumulation of NO_3_^−^ and phosphate (PO_4_^3−^) in vacuole to sustain plant growth^25^.

A constant decline of water resources, due to climate change, is one of the major threats to the future food security of ever increasing world population. The challenge of water scarcity is more urgent than ever: acute water shortage pose serious threats to productivity of major food crops; decreasing water flows put us in seriously water-scarce countries. The frequent shortage of water and deterioration of eco-environment due to progressive global climate change have greatly influenced agricultural production in arid and semi-arid regions of the world. Adoption of drought mitigation approaches such as selection for physiologically efficient S fertilizers may have value in management programs aimed at improving drought stress tolerance to increase grain yield of food crops like maize. In this study, we hypothesized that S induced improvement in drought tolerance may be attributed to increased photosynthetic activity and activation of antioxidant machinery. To test this hypothesis, we firstly optimized doses for S fertilization in maize seedlings that were later used in second experiment to evaluate the effects of S on physiological and biochemical processes of maize under drought stress.

## Materials and Methods

### Experimental material and conditions

Two pot experiments were conducted in wire house of MNS-University of Agriculture, Multan (MNS-UAM), Pakistan using completely randomized design with factorial arrangement and three replications. Seeds of indigenous maize hybrid viz. P1574 characterized as drought sensitive by Majeed *et al*.^26^ were obtained from local seed dealer of Pioneer Pakistan Pvt. Ltd. It is a highly digestible spring maize hybrid also used for silage purposes by local farmers. The seeds were initially treated with recommended doses of Topsin-M-70-WP (fungicide) and Imidacloprid (insecticide) for disinfection. The pots were filled with sandy loam soil collected from research field area of MNS-UAM. Before filling the pots, soil samples were randomly taken from collected soil to determine the physicochemical characteristics following the procedure reported by Jackson and Barak^27^ (Table 1).

**Table 1:** Physicochemical characteristics of soil used for the pot experiments.

Physical Characteristics		Chemical Characteristics					
Texture	Saturation percentage	pH	Organic matter (%)	Nitrogen (mg kg^−1^)	Phosphorus (mg kg^−1^)	Potassium (mg kg^−1^)	Sulfur (mg kg^−1^)
Sandy loam	24	8.1	0.79	102	8.50	240	2.1

### Pot experiment-I

First pot experiment was carried out to optimize sulfur (S) dose for selected maize hybrid using different S sources. Randomly selected healthy, uniform ten seeds were sown in plastic pots of 10 kg capacity of soil (25 cm diameter × 45 cm length). The seedlings were thinned later and only five seedlings were maintained after emergence in each pot. Nutrient solutions containing N, P and K were applied as fertilizers at the start of the experiment using urea (0.6 kg per pot), diammonium phosphate (0.3 kg per pot) and potassium oxide (0.2 kg per pot). Sulfur (S) fertilization was done one week after seedling emergence (V4, four leaf stage) through fertigation using various sources of S viz. K_2_SO_4_, CuSO_4_, FeSO_4_ and Na_2_SO_4_ applied at different rates of 15, 30 and 45 kg ha^−1^ (75, 150 and 225 mg per pot). All pots were weighed daily to estimate water lost through evapotranspiration and supplied with required amount of water. After five weeks of seedling emergence (V10), the seedlings were harvested for estimation of biomass attributes and later dried in an oven at 65 °C for at least 72 h to record dry weight and NPK analysis.

### Pot experiment-II

The second pot experiment was conducted to evaluate the effects of optimized S doses on physiological and biochemical processes of maize under drought stress. Ten seeds of same maize hybrid were sown in earthern pots (diameter 45 cm × length 60 cm) filled with 24 kg soil. Only three seedlings per pot were maintained after emergence, which were later reduced to only one healthy seedling in each pot. Recommended doses of P and K (80 kg ha^−1^ each) and 1/8^th^ N (120 kg ha^−1^) were fertigated at the time of sowing using diammonium phosphate, potassium oxide (0.96 g per pot) and urea (0.18 g per pot), whereas remaining N was applied at in three equal split doses of 0.42 g per pot as described by Naeem *et al*.^28^.

### Drought stress and sulfur fertilization

Drought stress was imposed one week after seedling emergence by keeping one set of plants (normal plants) at 100% water holding capacity (WHC), whereas water stressed plants were kept at 30% WHC based on gravimetric method as described by Nachabe^29^. The soil moisture content was measured daily using soil moisture meter ML-3 Theta Probe (Delta-T Devices, United Kingdom) and maintained by adding the amount of water lost through evapotranspiration.

Sulfur fertilization was done through fertigation, before initiation of drought stress, using optimized doses of K_2_SO_4_ (30 kg ha^−1^), Na_2_SO_4_ (30 kg ha^−1^), CuSO_4_ (45 kg ha^−1^) and FeSO_4_ (45 kg ha^−1^). The youngest mature leaves from each experimental unit were selected for the estimation of water status, SPAD value and activity of antioxidative enzymes at tasseling (VT) stage. The plants were harvested at physiological maturity and data regarding yield attributes was recorded from harvested plant material following standard procedures.

### Determination of NPK content

The above ground plant material including leaves (0.5 g) was dried in an oven and later grounded using Willey mill. The dried material was used for the determination of N, P and K content following the method described by Wolf^30^. Briefly, the powdered plant tissue was acid-digested with 5 ml of H_2_SO_4_ using BD50 digestion block (SEAL Analytical, Malaysia). Then 2 ml of H_2_O_2_ was added in tubes and heated at 350 °C for three and half hours until fumes were produced. Volume of extract was maintained by adding distilled water up to 50 ml in volumetric flask. The extract was filtered with Whatman-40 filter paper and N was determined using Kjeldhal method. The vanadium molybdate yellow colorimetric method was used for P determination, whereas K content was assayed using flame photometer (Sherwood M410, UK).

### Estimation of leaf relative turgidity and SPAD value

The detached youngest, fully expanded leaf was weighed immediately to record fresh weight (FW) and then dipped in distilled water for 24 h at 4 °C. Later, the leaves were taken out from distilled water, wiped with tissue paper and weighed to determine turgid weight (TW), Then same leaves were placed in an oven for 72 h at 65 °C to record dry weight (DW). Leaf relative turgidity (RT) was measured using following formula reported by Barrs^31^:

$${\rm{RT}}=[({\rm{FW}}-{\rm{DW}})/({\rm{TW}}-{\rm{DW}})]\times 100$$

The fully expanded young leaves were used to estimate leaf chlorophyll content expressed as SPAD value. The observations were made early in the morning between 9.00 and 11.00 a.m. using chlorophyll meter (SPAD-502, Minolta Corp.).

### Gas exchange measurements

The gas exchange characteristics were measured with a CIRAS-3 portable open-flow gas exchange system (PP Systems, Amesbury, USA). The system was used to record net photosynthetic rate (*A*), transpiration rate (*E*), stomatal conductance (*g*_s_) and sub-stomatal conductance (*C*_i_) of uppermost fully expanded leaf of each seedling. The chamber was adjusted at 100 mL min^−1^ mL airflow rate, 1200 μmol∙m^−2^∙s^−1^ density of photosynthetic photon flux, 390 ± 5 μmol∙mol^−1^ CO_2_ concentration rate, 99.9 kPa atmospheric pressure.

### Assay of antioxidative enzymes

Leaf samples were homogenized (1:5) in pestle and mortar using 50 mM Na_2_HPO_4_, pH 7.0 containing 1 M Sodium chloride,1 mM EDTA and 1% polyvinylpyrroldone. Enzyme activities were determined using supernatant of enzyme extract (EE) produced from sample solution after centrifugation (20,000 × g, 15 min) at 4 °C.

Catalase activity (CAT) activity was determined by monitoring the degradation of hydrogen peroxide (H_2_O_2_) according to Chance and Maehly^32^. Enzyme extract (200 µL) was added in reaction mixture (1.8 mL), which contained H_2_O_2_ (30 mM) and K-P-buffer (50 mM) of 7.0 pH. The decline in H_2_O_2_ was estimated as a reduction in optical density at 240 nm.

The procedure reported by Urbanek *et al*.^33^ was followed to estimate guaiacol peroxidase (GPX) activity. The reaction mixture (2 mL) was prepared by mixing 50 mM K-P buffer (pH 6.8) with H_2_O_2_ (20 mM) and guaiacol (20 Mm). The mixture was incubated at room temperature for 10 min. The reaction was stopped by adding 0.5 mM H_2_SO_4_ (5%) and absorbance was measured at 480 nm.

The enzymatic activity of superoxide dismutase (SOD) was recorded following the method of Van Rossun *et al*.^34^. The enzyme extract (50 µL) containing 50 mM K-P-buffer (pH 7.8). was added to 2 μM riboflavin, 75 μM nitroblue tetrazolium chloride (NBT), 100 μM EDTA and 13 mM L-methionine. The reaction was initiated in a chamber under illumination of a 30 W-fluorescent lamp for 10 min. The blue color formazane, produced as a result of photoreduction caused by nitroblue tetrazolium (NBT), was noted as increase in absorbance at 560 nm. No enzyme extract was added in reaction mixture used as control and kept in the dark. One SOD unit was defined as the quantity of enzyme required to inhibit 50% photoreduction of the NBT.

### Economic analysis

A benefit-cost analysis was carried out to conclude the economic feasibility of various sulfate fertilizers to alleviate the drastic influence of drought stress in maize. The rate of each S fertilizer i.e. K_2_SO_4_, FeSO_4_, CuSO_4_ and Na_2_SO_4_ used in this experiment was 30, 45, 45 and 30 kg ha^−1^ respectively. The cost of K_2_SO_4_, FeSO_4_, CuSO_4_ and Na_2_SO_4_ was 176, 1200, 1200 and 65 kg^−1^ in PKR (Pakistani rupees) respectively. Land preparation, sowing seed, irrigation, fertilizing, plant protection measures (insecticide, herbicide), harvesting and threshing was included in fixed cost. The gross income was estimated by using prevailing average marketing price in Pakistan, PKR 900 per 40 kg.

### Statistical analysis

All collected data were analyzed statistically using Fisher’s Analysis of Variance (ANOVA) technique on computer programme Statistix (version 9.1). The treatment means were compared using Tukey’s *post-hoc* test at 0.05 probability level.

## Results

### Biomass accumulation

Application of S fertilizers significantly (*P* < 0.01) influenced biomass attributes i.e. shoot length (SL), root length (RL), shoot fresh weight (SFW), root fresh weight (RFW), shoot dry weight (SDW) and root dry weight (RDW) of maize seedlings (Suppl. Table 1). Maize seedlings fertilized with K_2_SO_4_ at 30 kg ha^−1^ exhibited the highest increase in SL (119.24%) RL (71.08%), SFW (124.46%), RFW (68.59%), SDW (136.17%) and RDW (62.43%) compared to control (no S supply). Higher concentration of K_2_SO_4_ and Na_2_SO_4_ significantly (*P* < 0.01) reduced biomass accumulation, whereas application of CuSO_4_ and FeSO_4_ at 45 kg ha^−1^ gave maximum values for these attributes with respect to no S supply (Table 2).

**Table 2:** Biomass attributes of maize seedlings applied with various sources of sulfur fertilizers viz. K_2_SO_4_, FeSO_4_, CuSO_4_ and Na_2_SO_4_ at 0, 15, 30 and 45 kg ha^−1^. Shoot length = SL, Root length = RL, Shoot fresh weight = SFW, Root fresh weight = RFW, Shoot dry weight = SDW and Root dry weight = RDW. Different alphabets represent significant difference between mean values ± standard error.

Observations	K_2_SO_4_ (kg ha^−1^)				FeSO_4_ (kg ha^−1^)				CuSO_4_ (kg ha^−1^)				Na_2_SO_4_ (kg ha^−1^)			
	0	15	30	45	0	15	30	45	0	15	30	45	0	15	30	45
**SL**	26.0 ± 1.76 ^**g**^	39.7 ± 4.17^**c–f**^	57.0 ± 2.12^**a**^	54.3 ± 3.9^**ab**^	28.3 ± 1.8^**fg**^	38.3 ± 2.65^**c-f**^	40 ± 1.8^**c-f**^	46.7 ± 2.37^**a-c**^	31.0 ± 1.17^**e-g**^	31.0 ± 1.17^**e-g**^	39.0 ± 1.17^**c-f**^	43.0 ± 2.7^**b-e**^	30.0 ± 1.15^**fg**^	38.6 ± 1.5^**c-f**^	44.7 ± 4.5^**b-d**^	32.7 ± 2.06^**d-g**^
**RL**	27.7 ± 2.44^**c**^	36.0 ± 3.57^**a-c**^	47.3 ± 6.12^**a**^	45.0 ± 4.07^**ab**^	25.66 ± 1.48^**c**^	36.3 ± 1.8^**a-c**^	34.3 ± 2.96^**a-c**^	35.3 ± 2.37^**a-c**^	29.0 ± 0.58^**c**^	28.0 ± 1.17^**c**^	34.0 ± 1.76^**a-c**^	34.0 ± 2.56^**a-c**^	28.7 ± 0.89^**c**^	33.7 ± 1.48^**a-c**^	39.0 ± 4.11^**a-c**^	31.3 ± 1.79^**bc**^
**SFW**	2.60 ± 0.38^**bc**^	3.40 ± 0.35^**bc**^	5.82 ± 0.38^**a**^	5.82 ± 0.45^**a**^	2.64 ± 0.32^**bc**^	3.40 ± 0.53^**bc**^	3.66 ± 0.17^**bc**^	4.30 ± 0.54^**ab**^	2.34 ± 0.06^**c**^	3.61 ± 0.33^**bc**^	3.61 ± 0.26^**bc**^	4.30 ± 0.36^**bc**^	2.69 ± 0.23^**bc**^	3.61 ± 0.27^**bc**^	3.91 ± 0.32^**bc**^	3.51 ± 0.27^**bc**^
**RFW**	2.07 ± 0.08^**fg**^	2.29 ± 0.08^**e-g**^	3.49 ± 0.07^**ab**^	3.27 ± 0.06^**bc**^	2.11 ± 0.09^**e-g**^	2.40 ± 0.12^**e-g**^	2.88 ± 0.10 ^**cd**^	3.75 ± 0.12^**a**^	2.15 ± 0.09^**e-g**^	2.28 ± 0.07^**e-g**^	2.57 ± 0.08^**de**^	3.21 ± 0.11^**bc**^	2.05 ± 0.10 ^**g**^	2.53 ± 0.08^**d-f**^	3.10 ± 0.10^**bc**^	2.96 ± 0.09 ^**cd**^
**SDW**	0.47 ± 0.04^**c**^	0.54 ± 0.02^**c**^	1.11 ± 0.22^**a**^	0.97 ± 0.10^**ab**^	0.50 ± 0.06^**c**^	0.55 ± 0.03^**bc**^	0.58 ± 0.05^**bc**^	0.67 ± 0.02^**bc**^	0.54 ± 0.04^**c**^	0.58 ± 0.05^**bc**^	0.59 ± 0.06^**bc**^	0.71 ± 0.03^**a-c**^	0.44 ± 0.05^**c**^	0.71 ± 0.06^**a-c**^	0.83 ± 0.03^**a-c**^	0.60 ± 0.05^**bc**^
**RDW**	1.04 ± 0.03^**d**e^	1.19 ± 0.04^**c-e**^	1.77 ± 0.09^**a**^	1.72 ± 0.09^**a**^	0.96 ± 0.11^**e**^	1.08 ± 0.06^**de**^	1.35 ± 0.07^**b-d**^	1.75 ± 0.08^**a**^	1.04 ± 0.04^**de**^	1.14 ± 0.07^**de**^	1.32 ± 0.05^**b-e**^	1.56 ± 0.06^**ab**^	1.02 ± 0.08^**de**^	1.15 ± 0.08^**de**^	1.55 ± 0.06^**a-c**^	1.57 ± 0.06^**ab**^

### NPK content

**Figure 1:**
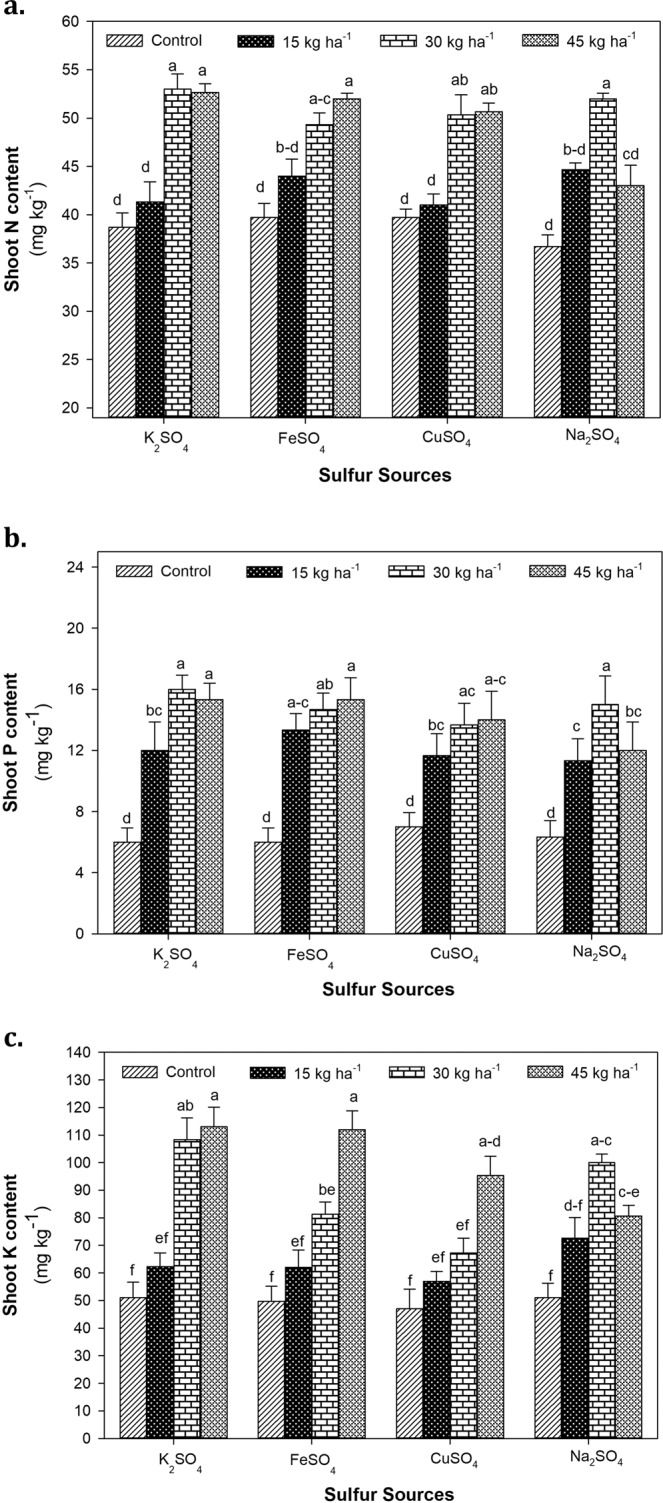
**(a)** The nitrogen (N), **(b)** phosphorous (P) and **(c)** potassium (K) content of maize seedlings affected by the application of various doses (0, 15, 30 and 45 kg ha^−1^) of sulfate fertilizers (K_2_SO_4_, FeSO_4_, CuSO_4_ and Na_2_SO_4_). The mean values with different letters indicate significant difference (*P* ≤ 0.05), according to *post hoc* Tukey’s test.

Fertigation with various S sources markedly (*P* < 0.01) affected NPK accumulation in shoots of maize seedlings (Suppl. Table 1). Excess S supply significantly (*P* < 0.001) improved N content and resulted in mean maximum N accumulation in seedlings treated with K_2_SO_4_ (53.67 mg kg^−1^), FeSO_4_ (53.0 mg kg^−1^) and CuSO_4_ (51.50 mg kg^−1^) at 45 kg ha^−1^. However, high dose of Na_2_SO_4_ i.e. 45 kg ha^−1^ reduced N content by 23% compared to 30 kg ha^−1^ that exhibited maximum N accumulation (52.0 mg kg^−1^) in shoot (Fig. 1a). Similar trend was noted for shoot P and K content as application of K_2_SO_4_, FeSO_4_ and CuSO_4_ at high S dose of 45 kg ha^−1^ significantly (*P* < 0.05) improved P accumulation by 53, 51 and 48% in comparison with no S supply, whereas mean maximum P content (15.25 mg kg^−1^) using Na_2_SO_4_ was recorded at 30 kg ha^−1^ and it declined significantly by 22% at higher dose of 45 kg ha^−1^ (Fig. 1b). Similarly, relative to no S treatment, maize seedlings fertigated with K_2_SO_4_, FeSO_4_ and CuSO_4_ at 45 kg ha^−1^ exhibited 56, 54 and 49% higher K content in shoot. In maize seedlings treated with Na_2_SO_4_, the highest K content (102.50 mg kg^−1^) was recorded at 30 kg ha^−1^ in comparison with 45 kg ha^−1^ that considerably reduced shoot K content by 17% (Fig. 1c).

### Leaf relative turgidity and SPAD value

**Figure 2:**
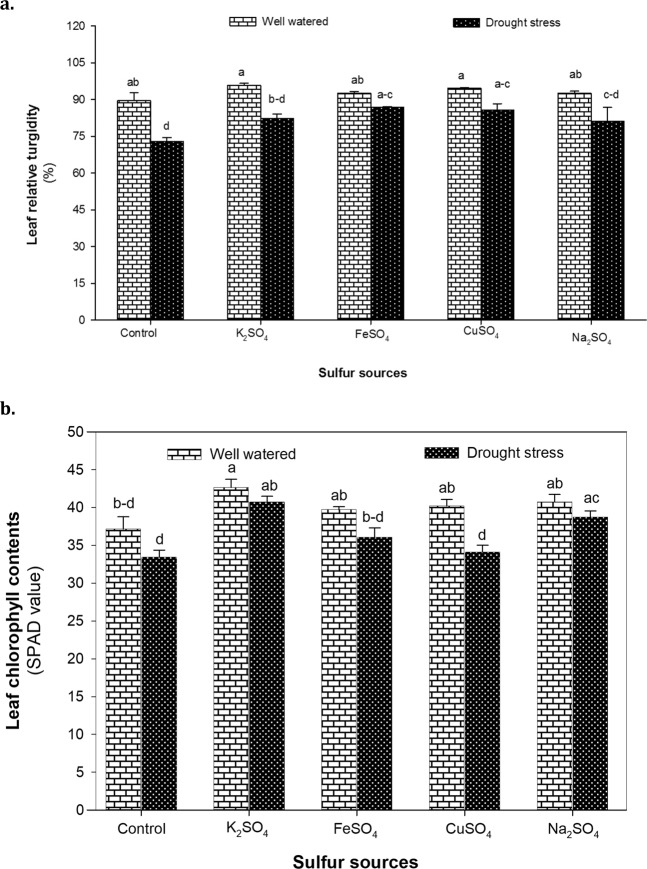
**(a)** The leaf relative turgidity (RT) and **(b)** the chlorophyll content (Chl) of maize plants affected by the application of optimized doses of sulfate fertilizers (K_2_SO_4_, FeSO_4_, CuSO_4_ and Na_2_SO_4_) under normal (100% WHC) and drought stress (30% WHC) conditions. The mean values with different letters indicate significant difference (*P* ≤ 0.05), according to *post hoc* Tukey’s test.

The main effects of drought stress (D) and sulfur sources (S) were found significant (*P* < 0.01) for leaf relative turgidity (RT) and chlorophyll content (Chl) (Suppl. Table 2). Drought stress caused a substantial decline (*P* < 0.01) in RT and Chl of maize seedlings by 13 and 10%, respectively. Application of S fertilizers significantly (*P* < 0.01) ameliorated the drastic effects of drought stress. Maize plants treated with FeSO_4_ and CuSO_4_ maintained the highest RT i.e. 86.78 and 85.68%, respectively under water deficit conditions. Similarly, K_2_SO_4_ and Na_2_SO_4_ application increased RT by 12 and 10% compared to no S supply in maize under drought stress (Fig. 2a). The highest Chl was recorded in maize plants fertilized with K_2_SO_4_ under normal (42.67 SPAD value) as well as drought stress (40.73 SPAD value) conditions. Interestingly, CuSO_4_ application negatively influenced Chl and resulted in the lowest values along with control in both normal (37.17 SPAD value) and water stressed (31.78 SPAD value) maize plants (Fig. 2b).

### Gas exchange characteristics

**Figure 3:**
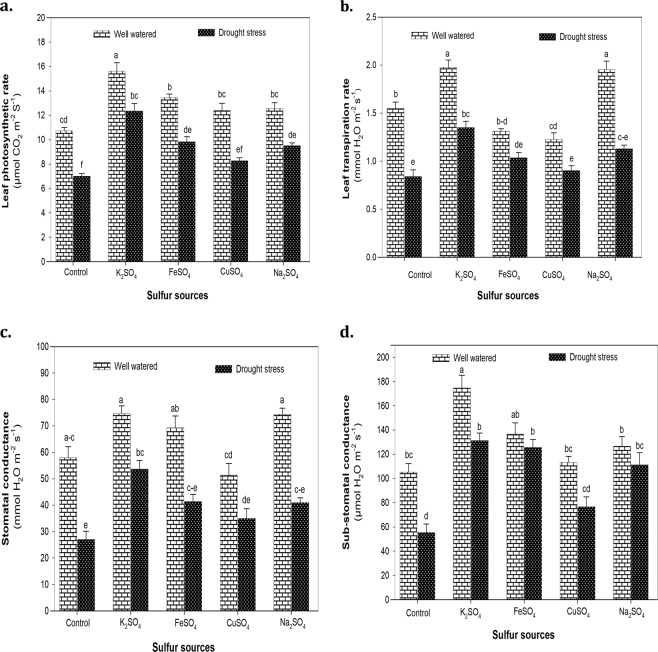
**(a)** The leaf photosynthetic rate (*A*), **(b)** transpiration rate (*E*), **(c)** stomatal conductance (*g*_*s*_) and (**d**) sub-stomatal conductance (*C*_*i*_) of maize plants affected by the application of optimized doses of sulfate fertilizers (K_2_SO_4_, FeSO_4_, CuSO_4_ and Na_2_SO_4_) under normal (100% WHC) and drought stress (30% WHC) conditions. The mean values with different letters indicate significant difference (*P* ≤ 0.05), according to *post hoc* Tukey’s test.

Exposure to drought stress considerably (*P* < 0.001) reduced *A* (37.75%), *E* (52.47%), *g*_*s*_ (31.24%) and *C*_*i*_ (65.49%) of maize plants compared to normal conditions, irrespective of S application (Suppl. Table 2). Exogenous S fertilization with K_2_SO_4_ resulted in the highest increase in *A* (43%) and *E* (61%) of maize plants subjected to drought stress (Fig. 3a,b). A marked increase of 43 and 23% in *A* and *E*, respectively was also observed by FeSO_4_ application under water deficit conditions (Fig. 3a,b).

The regulation of stomatal apparatus was also significantly (*P* < 0.001) influenced by the application of various S fertilizers (Suppl. Table 2). Application of K_2_SO_4_ effectively improved *g*_*s*_ and *C*_*i*_ of maize plants by 98 and 127%, respectively under drought stress. Fertilization with FeSO_4_ and Na_2_SO_4_ also increased *g*_*s*_ by 53% (Fig. 3c), whereas application of Na_2_SO_4_ enhanced *C*_*i*_ by 105% under water deficit conditions (Fig. 3d).

### Antioxidant enzyme activities

**Figure 4:**
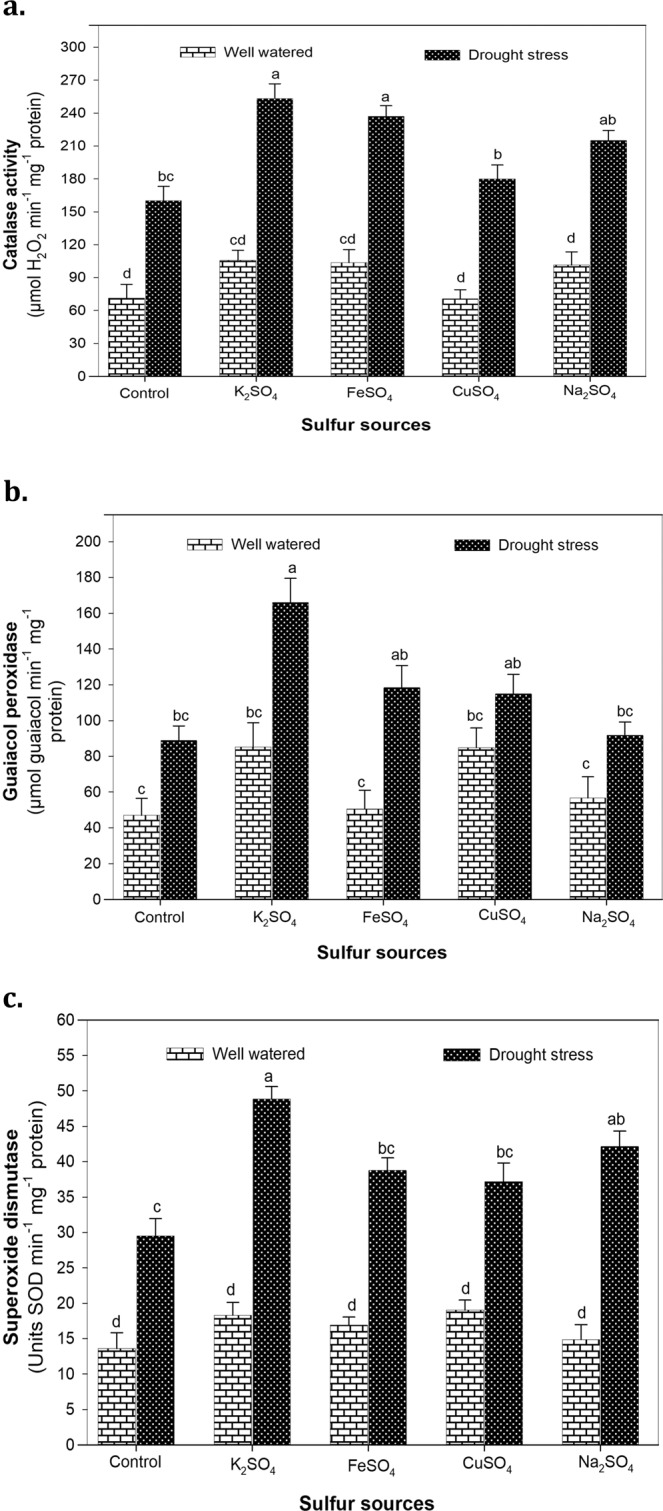
**(a)** The catalase (CAT), **(b)** guaiacol peroxidase (GPX) and **(c)** superoxide dismutase (SOD) activity of maize plants affected by the application of optimized doses of sulfate fertilizers (K_2_SO_4_, FeSO_4_, CuSO_4_ and Na_2_SO_4_) under normal (100% WHC) and drought stress (30% WHC) conditions. The mean values with different letters indicate significant difference (*P* ≤ 0.05), according to *post hoc* Tukey’s test.

Drought stress markedly (*P* < 0.001) markedly increased the activities CAT (131%), GPX (79%) and SOD (137%) compared to well-watered conditions (Suppl. Table 2). The highest CAT activity (253.33 μmol H_2_O_2_ min^−1^ mg^−1^ protein) was noted in leaves of water stressed maize plants supplemented with K_2_SO_4_ and did not differ significantly from FeSO_4_ (237.0 μmol H_2_O_2_ min^−1^ mg^−1^ protein) and Na_2_SO_4_ (215.0 μmol H_2_O_2_ min^−1^ mg^−1^ protein) (Fig. 4a). Similarly, the maize plants treated with K_2_SO_4_, FeSO_4_ and Na_2_SO_4_ exhibited 87, 34 and 30% higher GPX activity, respectively in comparison to plants with no S application (control) (Fig. 4b). Application of S fertilizers effectively enhanced SOD activity (*P* < 0.001) and gave the maximum increase (65%) in plants supplemented with K_2_SO_4_ under water deficit conditions. Likewise, Na_2_SO_4_, FeSO_4_ and CuSO_4_ upregulated SOD activity by 43, 31 and 29% compared to control under drought stress (Fig. 4c).

### Yield and yield components

**Figure 5:**
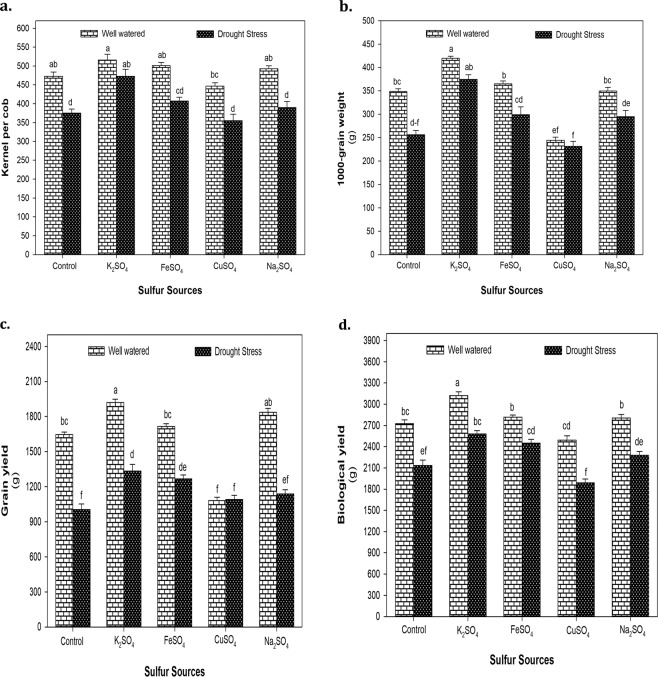
**(a)** The number of kernels per cob (KC), **(b)** 1000-grain weight (GW), **(c)** grain yield (GY) and **(d)** biological yield (BY) of maize plants affected by the application of optimized doses of sulfate fertilizers (K_2_SO_4_, FeSO_4_, CuSO_4_ and Na_2_SO_4_) under normal (100% WHC) and drought stress (30% WHC) conditions. The mean values with different letters indicate significant difference (*P* ≤ 0.05), according to *post hoc* Tukey’s test.

The main effects of D and S were significant (*P* < 0.01) for all maize yield attributes viz. kernels per cob (KC), 1000-grain weight (GW), grain yield (GY) and biological yield (BY), however, significant (*P* < 0.05) two way interaction (D × S) was only observed for KC, GW and GY (Suppl. Table 3). Exposure to drought stress considerably reduced KC, GW, GY and BY by 24, 19, 23 and 41%, respectively with respect to normal conditions. The highest increase in KC (41%) and GW (27%) was recorded by K_2_SO_4_ application compared to control (no S supply) under drought stress conditions (Fig. 5a,b). Interestingly, CuSO_4_ application reduced GW by 11% in water stressed maize plants compared to normal ones (Fig. 5b). Similar trend was observed for GY and BY as K_2_SO_4_ supply significantly increased GY by 17 and 33% compared to control under normal and water deficit conditions, respectively (Fig. 5c). Likewise, it improved BY by 15 and 21% in normal and water stressed maize plants (Fig. 5d). A marked increase in GY and BY was also observed by FeSO_4_ (26 and 15%) and Na_2_SO_4_ (13 and 7%) application under drought stress conditions (Fig. 5c,d).

Application of various sulfate fertilizers improved the net benefit cost ratio, however, high market cost of FeSO_4_ resulted in negative net income. Similarly, CuSO_4_ and Na_2_SO_4_ induced toxicity also negatively influenced the net benefit ratio, whereas K_2_SO_4_ application was found to be most economical for improving maize yield under water deficit conditions (Table 3).

**Table 3:** Effect of various sulfate fertilizers application on net income and benefit-to cast ratio of maize under normal and drought conditions.

Treatment	Total expenditure (^*^PKR ha^−1^)		Grass income (PKR ha^−1^)		Net Income (PKR ha^-1^)		Benefit: cost ratio	
	Normal	Drought	Normal	Drought	Normal	Drought	Normal	Drought
Control	134394	132754	174020	97900	39626	−34854	1.29	0.74
K_2_SO_4_	139794	138154	229526	166100	89732	27946	1.64	1.20
FeSO_4_	188394	186754	208560	143726	20166	−43028	1.11	0.77
CuSO_4_	188394	186754	155540	95700	32854	−91054	0.83	0.51
Na_2_SO_4_	137394	135754	185174	121000	47780	−14754	1.35	0.89

*1.00 USD = 159.7 PKR.

## Discussion

This study presents data to interpret firstly the effects of various S fertilizers and their doses on biomass accumulation and NPK uptake in maize seedlings. Secondly, the physiological and biochemical significance of S fertilizers in drought stress tolerance, with a particular focus on regulation of gas exchange characteristics and enzymatic antioxidants to improve maize yield will be discussed. Exogenous S application significantly (*P* < 0.01) enhanced SL, RL, SFW, RFW, SDW and RDW of maize seedlings (Suppl. Table 1). As biomass attributes were increased by S fertilization, it may be concluded that S availability enhances photosynthetic rate and stimulates translocation of photosynthates towards sink^22^. However, this stimulating effect of S fertilizers was found to be dose dependent and considerable variation was observed among various doses (Table 2). High doses of K_2_SO_4_ and Na_2_SO_4_ (45 kg ha^−1^) showed adverse effects on biomass accumulation as reported earlier in studies involving maize^35^ and *Brassica rapa*^36^. The possible general explanation for this reduction may be the altered toxicity of sulfate anion (SO_4_^2−^) by the presence of Na^+^^37^.

Addition of sufficient amount of nutrients is first agricultural measure to increase crop performance^38^, however, the uptake of mineral nutrients is considerably influenced by the interactions between metal ions at different physiological levels^24^. This is particularly true for S as drought stress results in low SO_4_^2−^ absorption and its subsequent translocation to leaves that also limits NO_3_^−^ translocation, consequently reducing nitrogen use efficiency^39^. Similarly, S-deprivation influences K accumulation in shoots providing evidence that K^+^ acts as counter-cation in the absence of SO_4_^2−^ in leaf tissue^24,40^. The results in present study showed that low water availability considerably (*P* < 0.01) restricts NPK accumulation in shoots of maize seedlings (Fig. 1a–c). Drought induced restriction in nutrient accumulation might be associated to reduced *E*, resulting in limited root absorbing power to uptake N, P and K^41^. Drought stress drastically influences maize at early growth stages by changing root maintenance system and architecture^42^. Recent studies by Studer *et al*.^43^ confirmed that the seedling growth of maize is most sensitive to nutrient deficiency, particularly under drought stress conditions. In agreement with the findings of Raza *et al*.^22^ in sesame, our results showed that S fertilization significantly (*P* < 0.05) increased NPK content in shoots of maize seedlings (Suppl. Table 1), this may be associated with improved nutrient acquisition and utilization of these macronutrients with S application. Shoot N content were considerably (*P* < 0.001) increased by S application (Fig. 1a) providing further evidence that these nutrients are highly inter-related^32^ and significantly influence protein metabolism, thereby quality of crop plants^44,45^. Lošák *et al*.^46^ found positive correlation between N and S to increase camelina seed yield in S deficient soils. They suggested that combined S and N application could be utilized as an effective approach to improve oil and protein yield. High concentration of these nutrients obtained by K_2_SO_4_ might also be attributed to presence of K^+^ considering the importance of this cation to improve drought tolerance in crop plants^47,48^. Previous studies on *Eruca sativa*^49^ sesame^50^ and maize^51^ also reported an increased P and K content by S application. It may be inferred that S fertilization after biochemical oxidation produces H_2_SO_4_, which improves nutrient availability to plants^52,53^. Recent report of Reich *et al*.^31^ showed that S deficiency reduced K^+^ accumulation in shoots of Chinese cabbage highlighting the role of S in xylem loading and translocation of K^+^ to the shoot. Positive effect of FeSO_4_ on nutrient accumulation in maize seedlings (Fig. 1a-c) suggests the cooperative role of S and Fe in plant metabolism, for example Fe-S clusters in the electron transport chain^54^. Cross talk between S and Fe uptake and metabolism in plants is particularly important because S deficiency not only limits the synthesis of Fe-S cluster proteins but also reduces the translation in general^55^.

Maintenance of leaf RT and Chl indicate plant ability to tolerate water stress conditions^28,56^. In the present study, drought induced reduction (*P* < 0.001) in leaf RT (Suppl. Table 2) might be the result of loss in turgor or impaired photosynthetic rate or combination of these factors^57,58^. Leaf RT is often used as a criterion to determine the degree of drought stress as loss in RT leads to protoplasm dehydration^59^ and decrease in cell enlargement and expansion^60^. Application of S fertilizers significantly (*P* < 0.01) enhanced leaf RT (Fig. 2a) indicating that S availability restricts the movement of ions and solutes in the cells associated with reduced osmotic potential under water deficit conditions^49^. High RT by S application might be associated with increased water uptake by roots as drought stress reduces translocation of newly absorbed SO_4_^2−^ to shoot also reported in *B. napus*^61^.

Biosynthesis and maintenance of photosynthetic pigments is considered to be a potential indicator of drought tolerance in crop plants^4^. Measurement of total Chl using chlorophyll meters such as SPAD-502 is an effective, non-destructive and inexpensive method that provides absolute values of Chl per unit leaf area^62^. A marked decline in SPAD value (*P* < 0.001) was noted in S deficient maize plants (control) under drought stress (Fig. 2b), which may be due to decrease in S compounds such as Cys and Met that serve as integral component of chloroplast targeted proteins. Our findings are in line with reports of Houhou *et al*.^63^ in *E. sativa* and Kassem *et al*.^64^ in *Lycopersicum esculentum* providing further evidence that S starvation influences the coordination between light reaction and Calvin cycle in chloroplast ultimately reducing Chl in leaves. In rice, Lunde *et al*.^65^ found a significant decrease in Chl of S deficient plants followed by a noteable reduction in photochemical performance and decreased photosynthetic activity. Maize seedlings supplemented with CuSO_4_ exhibited a significant (*P* < 0.001) decrease in Chl (Fig. 2b) that might be attributed to Cu ion toxicity leading to ultra-structural alterations and photochemical oxidation in chloroplast^66^. Excess Cu inhibits carboxylase activity and causes swelling of thylakoids as reported by Ibrahim *et al*.^67^ in *Gynura procumbens*.

The reduction in gas exchange attributes viz. *A*, *E*, *g*_*s*_ and *C*_*i*_ is generally considered to be the first effects of drought stress due to chlorophyll degradation and restriction in available CO_2_^68,69^. Reduced gas exchange under S deficient conditions has been previously reported in several plant species such as barley^70^, mustard^71^ and rape^72^. In this study, application of S fertilizers considerably (*P* < 0.001) increased *A* (Fig. 3a) suggesting that S availability improves CO_2_ assimilation and protein abundance to alleviate drought induced inhibition of photosynthetic capacity in maize plants. Adequate S-supplementation favors formation of S containing amino acids (Cys and Met) and reduces oxidative stress (high CAT, GPX and SOD activity) to stabilize Rubisco and thylakoid membrane proteins under drought stress conditions^14^. Compared to no S supply, application of K_2_SO_4_ and FeSO_4_ markedly (*P* < 0.001) enhanced *g*_*s*_ and *C*_*i*_ (Fig. 3c,d) accompanied by high *E* (Fig. 3b) suggesting that these fertilizers influence stomatal regulation and further stabilize Fe-S clusters to improve the functioning of vital cellular processes such as photosynthesis and respiration under water deficit conditions^73,74^. In a study involving contrasting *B. napus* genotypes (Mosa and Saturnin), Lee *et al*.^39^ reported higher photosynthetic activity in genotype (Saturnin) with high sulfur use efficiency that ultimately contributed to better drought tolerance.

Drought stress as well as S deprivation causes metabolic imbalance that leads to oxidative burst in plant cell^75^. S deficiency stimulates peroxidation of biomolecules due to excessive accumulation of reactive oxygen species (ROS) and reduced synthesis of S-containing compounds^76^. In contrast to no S supply, application of S fertilizers considerably (*P* < 0.001) increased the activities of CAT, GPX and SOD (Suppl. Table 2), which was consistent with the maintenance of photosynthetic capacity following S supplementation under drought stress as reported by Ma *et al*.^77^ in wheat. Adequate S supply helps to counteract the drastic effects of ROS on nucleic acids and proteins through upregulation of antioxidant enzymes such as CAT, GPX and SOD^78^. These antioxidative enzymes serve as scavengers of O_2_ and H_2_O_2_ and help to prevent the production of toxic HO̅̅̅̅̅^79^. It is evident from the results that S mediated high antioxidant activity (Fig. 4a–c) corresponds to drought tolerance in maize. The combined action of CAT and SOD converts highly toxic O_2_^−^ and H_2_O_2_ into molecular oxygen and water, respectively to prevent ROS induced cellular damage in plants^41,80^. The availability of S promotes photosynthetic assimilation of SO_4_^2−^ to produce Cys that may be used to synthesize Met or converted into glutathione to regulate protein and cell function^81^ under environmental stresses like drought^11^. Compared to other S sources, higher antioxidant activity by K_2_SO_4_ application is in agreement with the recent reports of Zareei *et al*.^82^ in black grapes and Marques *et al*.^83^ in eggplant suggesting the coordinated action of K^+^ and SO_4_^2^ to regulate photosynthesis and translocation of photosynthates from roots to shoots, thereby alleviating drought induced oxidative damage in maize plants.

The overall impact of drought stress on yield and yield attributes of maize plants was highly significant (*P* < 0.001) with no S supply (Suppl. Table 3) that might be associated with reduced translocation of sugars to developing kernels under water deficit conditions^84^. This poor supply of sugars starves embryo and induces abortion of ovary ultimately affecting grain formation and yield of maize^85^. Positive effects of S fertilization on yield (Fig. 5a-d) suggest that SO_4_^2^ availability stimulates movement of assimilates from phloem into the apoplast due to higher photosynthetic activity and enhanced stomatal regulation (also observed in present study) under drought stress conditions. In line with our findings, early reports of Dewal and Pareek^86^ and Shahzad *et al*.^47^ showed ameliorative effects of K_2_SO_4_ application to improve GY in water stressed wheat and maize, respectively. Our results also showed a significant effect of FeSO_4_ application on maize yield attributes (Fig. 5a-d) as reported by Farokhi *et al*.^87^ in sunflower and Heidari *et al*.^50^ in sesame. The possible explanation for this increased yield by S fertilization could be the involvement of S-containing compounds in vital physiological and biochemical processes to modulate stress response under water deficit conditions^88^. The reduction in GY by CuSO_4_ application might be due to toxic effects of Cu^2+^ on photosynthetic electron transport chain resulting in protein denaturation and deactivation of antioxidant enzymes in plant cell^89^.

## Conclusion

The present study concludes that S starvation has a diverse impact on physiological and biochemical processes with important implications for maize yield under drought stress conditions. In contrast, S availability positively influenced the leaf water status, gas exchange characteristics and antioxidative machinery in water stressed maize plants. Among various S sources, K_2_SO_4_ application resulted in the maximum increase in yield providing further evidence that K^+^ and SO_4_^2−^ are strongly correlated to improve yield in crop plants. A marked increase in growth and yield was also noted by FeSO_4_ fertigation indicating that application of this fertilizer influences vital cellular processes including synthesis of Fe-S cluster proteins to improve drought tolerance in maize. However, high market cost of this fertilizer resulted in negative cost:benefit ratio for this fertilizer. Similary, Na_2_SO_4_ supply improved maize yield but high dose of this fertilizer induces toxicity, which may be due to accumulation of Na^+^ in rhizosphere. Similarly, CuSO_4_ application causes toxicity and significantly reduced yield compared to other S fertilziers. Moreover, these fertilzers were not found to be economical for improving yield under drought stress conditions.

## Supplementary information

Supplementary Tables.
