# Supplementary material for: Sulfate-mediated Drought Tolerance in Maize Involves Regulation at Physiological and Biochemical Levels
Source: Sci Rep. 2020 Jan 24;10:1147. doi: 10.1038/s41598-020-58169-2 (PMC6981264; doi:10.1038/s41598-020-58169-2)
Supplement: Supplementary file 1 — Supplementary Tables. [file 41598_2020_58169_MOESM1_ESM.pdf]

Sulfate-mediated Drought Tolerance in Maize Involves Regulation at Physiological and Biochemical Levels

Muhammad Munir Usmani[1], Fahim Nawaz[1*], Sadia Majeed[2], Muhammad Asif Shehzad[1], Khawaja Shafique Ahmad[3], Gulzar Akhtar[4], Muhammad Aqib[1], Rana Nauman Shabbir[5]

[1]*Department of Agronomy, MNS University of Agriculture, Multan, Pakistan*

[2]*Department of Agronomy, University College of Agriculture and Environmental Sciences, Bahawalpur, Pakistan*
[3]*Department of Botany, University of Poonch, Rawalakot 12350, Pakistan*
[4]*Department of Horticulture, MNS University of Agriculture, Multan, Pakistan*
[5]*Department of Agronomy, Faculty of Agriculture, Bahauddin Zakariya University, Multan*

*Corresponding author's email:
fahim.nawaz@mnsuam.edu.pk/fahim5382@gmail.com

Suppl. Table 1: Summary of the ANOVA for shoot length (SL), root length (RL), shoot fresh weight (SFW), root fresh weight (RFW) and shoot dry weight (SDW), root dry weight (RDW), nitrogen (N), phosphorous (P) and potassium (K) content of maize seedlings applied with various sources of sulfate fertilizers viz. K_2SO_4, $FeSO_4$, $CuSO_4$ and Na_2SO_4 at 0, 15, 30 and 45 kg ha^{-1}.

SOV	SL (cm)	RL (cm)	SFW (g)	RFW (g)	SDW (g)	RDW (g)	N (mg kg^{-1})	P (mg kg^{-1})	K (mg kg^{-1})
Sulfur sources (S)	***	***	***	***	**	**	***	*	***
Sulfur doses (L)	***	***	***	***	**	***	NS	***	***
S × L	***	NS	***	***	***	*	**	*	***
CV	4.14	4.20	5.05	5.68	9.25	9.16	7.71	8.57	11.86

[a] SOV= source of variation
[b] coefficient of variation
NS = $P \geq 0.05$; * = $P \leq 0.05$; *** = $P \leq 0.001$

Suppl. Table 2: Summary of the ANOVA for leaf relative turgidity (RT), SPAD value (Chl), photosynthetic rate (*A*), transpiration rate (*E*), stomatal conductance (g_s), sub-stomatal conductance (C_i), catalase (CAT), guaiacol peroxidase (GPX) and superoxide dismutase (SOD) activity of maize with or without exposure to drought stress and applied with optimized doses of sulfate fertilizers viz. K_2SO_4, $FeSO_4$, $CuSO_4$ and Na_2SO_4.

SOV	RT (%)	Chl (SPAD value)	*A* (µmol CO_2 m^{-2} s^{-1})	*E* (mmol H_2O m^{-2} s^{-1})	g_s (mmol H_2O m^{-2} s^{-1}	C_i (µmol H_2O m^{-2} s^{-1})	CAT (µmol H_2O_2 min^{-1} mg^{-1} protein)	GPX (µmol guaiacol min^{-1} mg^{-1} protein)	SOD (Units SOD min^{-1} mg^{-1} protein)
Drought stress (D)	***	***	***	***	***	***	***	***	***
Sulfur sources (S)	**	***	***	***	***	***	***	***	***
D × S	NS	NS	***	***	***	***	NS	NS	***
CV	4.12	4.73	8.27	8.71	10.24	10.07	12.96	21.58	12.04

[a] SOV= source of variation
[b] coefficient of variation
NS = $P \geq 0.05$; * = $P \leq 0.05$; *** = $P \leq 0.001$

Suppl. Table 3: Summary of the ANOVA for kernels per cob (KC), 1000-grain weight (GW), grain yield (GY) and biological yield (BY) of maize with or without exposure to drought stress and applied with optimized doses of sulfate fertilizers viz. K_2SO_4, $FeSO_4$, $CuSO_4$ and Na_2SO_4.

SOV	KC	GW (g)	GY (g)	BY (g)
Drought stress (D)	***	***	***	***
Sulfur sources (S)	***	***	***	***
D×S	*	**	***	NS
CV	5.08	5.45	4.09	3.75

[a] SOV= source of variation
[b] coefficient of variation
NS = $P \geq 0.05$; * = $P \leq 0.05$; *** = $P \leq 0.001$
